# A cross-sectional study on adult lifestyle habits during the COVID-19 pandemic

**DOI:** 10.1371/journal.pone.0299668

**Published:** 2024-05-20

**Authors:** Ghadeer A. R. Y. Suaifan, Ala’ M. Abu-Odeh, Mayadah B. Shehadeh, Fahid Abu Jbara, Ward Abu Jbara, Razan I. Nassar

**Affiliations:** 1 Department of Pharmaceutical Sciences, School of Pharmacy, The University of Jordan, Amman, Jordan; 2 Department of Pharmaceutical Chemistry and Pharmacognosy, School of Pharmacy, Applied Science Private University, Amman, Jordan; 3 School of Medicine, The University of Jordan, Amman, Jordan; 4 School of Dentistry, The University of Jordan, Amman, Jordan; University of Petra (UOP), JORDAN

## Abstract

COVID-19 has spread and developed into a pandemic disease, forcing countries to impose challenging protocols and lockdowns. This study assessed shopping, food consumption behavior, and feelings in Jordan and several Arab countries during the COVID-19 pandemic. A cross-sectional web-based survey among the Middle East population was conducted using an online questionnaire between July and September 2022. Participants were requested to answer a standardized and validated structured questionnaire. Demographic information, shopping behavior information, and mental health data were requested. A total of 542 respondents were included in the study. During COVID-19 quarantine, participants (68.6%) reported decreased shopping frequency and buying more food than usual (37.5%). Cereals and legumes were the primary food types stored by participants (76.9%). Multiple logistic regression revealed the age of the participant as a significant factor affecting storing of food (being ≤ 25 years old (OR = 0.456, *p* value = 0.038)). 75.7% of female participants eat less frequently in restaurants than usual. In contrast, among males, 48.5% reported that they eat at restaurants less frequently than usual. The country of residency and gender were the significant factors affecting negative feelings and emotions. Participants in countries other than Jordan had a higher negative feeling score (Beta = 0.086, *p* value = 0.042). Furthermore, females had a higher negative feeling score (Beta = -0.128, *p* value = 0.003) as the negative feelings score for females was 3.58 (SD = 5.443). On the other hand, it was 2.10 (SD = 5.091) for males. The COVID-19 pandemic has altered Jordanians’ attitudes, shopping, and food consumption habits. Although positive behaviors have improved, such as shopping less frequently, eating home-cooked meals, and dining with family, frequent snacking and food storage have increased. Finally, public awareness of shopping and food consumption habits should be promoted.

## Introduction

Throughout history, human has encountered major pandemics that impacted people’s health, which increased individuals’ awareness of the importance of isolation and distancing themselves from infected individuals as a first line of defense [1]. Recently, in 2019, humanity confronted an unprecedented health crisis known as the coronavirus disease 2019 (COVID-19) [2] caused by the severe acute respiratory syndrome coronavirus 2 (SARS-CoV-2). The COVID-19 pandemic imposed many burdens, including a rise in the mortality rate, economic collapse, overwhelming health systems, and financial losses [3,4].

Prevention is the key to limiting the spread of coronavirus. The World Health Organization (WHO) endorsements have focused on preventing the spreading of the virus through good hygiene, social distancing, and only exiting home when necessary [5]. Therefore, governments around the globe implemented lockdowns and social distancing to prevent and control the spread of the coronavirus [3]. Consequently, universities and schools were postponed, work was suspended, and restrictions on outdoor mobility were imposed, with exceptions only for emergencies and essential needs [6].

The imposed restrictions affected the quality of life and daily habits. Many studies have investigated the individual’s lifestyle during this time, focusing on aspects such as diet, physical activity, patterns of sleep, and stress levels. It was found that the lockdown and other protective measures had negatively affected individuals’ lifestyle habits [7]. For example, decrease in physical activity, alternations in eating habits such as increased food consumption, rising rates of smoking and alcohol use, as well as alterations in psychological well-being. Therefore, this study aimed to assess the effects of the COVID-19 pandemic and the associated lockdown on adults’ eating habits and other behaviors [7].

## Method

### Study design and participants

A cross-sectional, web-based study design was utilized to assess lifestyle habits during the COVID-19 pandemic. The research was conducted between the 15^th^ of July and the 15^th^ of September, 2022 to explore the impact of the pandemic on adult lifestyle habits as people transitioned to a new normal. While it is true that lockdowns were mostly lifted during this timeframe, the effects of the pandemic on individuals’ behavior and lifestyle persisted beyond the immediate lockdown period. The inclusion criteria included adults aged 18 years and above, residing in Jordan or other Arab countries, active social-media users, capable of reading and understanding Arabic, and agreed to complete the questionnaire. Following the nondiscriminatory snowball sampling method, participants were encouraged to share the questionnaire with their family members, friends, and relatives.

The landing page of the questionnaire included a brief cover letter describing the aim and objectives of the study. Potential participants were requested to confirm their eligibility, understanding, and consent. The survey was created and structured using Google Forms^®^. It was disseminated via multiple social media platforms, such as email, WhatsApp, and Facebook, targeting potential adults with access to the online survey. The sampling method was chosen for its convenience and time-efficient. Ethical approval for the study was obtained from the Institutional Review Board (IRB) at The University of Jordan (IRB 4–2022).

### Sample size calculation

Sample size estimation at a 95% significance level and 5% error margin was calculated using OpenEpi, Version, and Kish formula15 using the equation below [4].


n=[DEFF*Np(1−p)]/[(d2/Z21−α/2*(N−1)+p*(1−p)]

Where,

N: Population size (for finite population correction factor or FPC)

P: Hypothesized % frequency of outcome factor in the population: 5%±5

d: Confidence limits as % of 100 (absolute ±%): 5%

DEFF: Design effect (for cluster surveys): 1

Z: Value for 95% confidence limits

p: Estimated prevalence

Accordingly, the ideal sample size should be 384. However, considering the non-response rate, 10% of the calculated sample size was added. Thus, the study’s final representative sample size was estimated to be 422 [8].

### Statistical analysis

Participants’ responses were coded and entered into a customized database using the Statistical Package for the Social Sciences (SPSS), Version 24.0 (IBM Corp., Armonk, New York, USA).

Participants were allocated to one of the two groups: Jordan and the other. Multiple logistic regression was used to explore the independent factors influencing storing food during the home quarantine period. Simple logistic regression was conducted as a first step to screen the potential variables for multiple logistic regression. Any variable with a *p* value < 0.250 was eligible to be entered into the multiple regression. It was guaranteed that the independent variables included in the multiple regression did not exhibit multicollinearity. Any variable in the multiple regression that has a *p* value of 0.05 or lower has been considered significant.

Seven independent factors affecting storing food during the quarantine were assessed in the multiple regression. Utilizing 20 participants for each independent factor (n = 7), 140 study participants would be needed to conduct the multiple regression. After considering that 5–20 participants are necessary for each independent factor, the study’s sample size is adequate and representative. Multiple logistic regression was also used to explore the independent factors influencing taking nutritional supplements to boost immunity among the study participants.

Multiple linear regression was used to assess the independent factors influencing the negative feeling score, which expresses the negative feelings that a person feels or the lack of positive feelings. Hence, the higher the score, the more negative a person feels. The negative feeling score was calculated for each participant, giving the negative feelings (n = 6; nervous, anxiety, depression, sadness, fear, and boredom) a score of +1 if the answer was ’Yes,’ on the other hand, a score of -1 was given for ’No’ answers. Regarding positive feelings (n = 4; calmness, optimism, happiness, and enthusiasm), a score of +1 was given for ’No’ responses, and a score of -1 was given for ’Yes’ responses. Consequently, the negative feeling score has a potential range from -10 to 10.

## Results

### Characteristics of participants

A total of 542 participants completed the questionnaire. 76% of the respondents were females, and 65.3% were between 18 and 25. 83% hold a bachelor or higher degree. Half the participants were students; most lived in Jordan (Table 1).

**Table 1:** Sociodemographic characteristics of the study’s participants (n = 542).

Sociodemographic Characteristics		n (percent)
**Age**	≤ 25 years old	354 (65.3)
	> 25 years old	188 (34.7)
**Gender**	Female	412 (76)
	Male	130 (24)
**Martial status**	Married	153 (28.2)
	Others (single, divorced, and widowed)	389 (71.8)
**Country**	Jordan	458 (84.5)
	Other*	84 (15.5)
**Education**	< Bachelor degree	91 (16.8)
	≥ Bachelor degree	451 (83.2)
**Profession**	Full/part-time job	127 (23.4)
	Not working	45 (8.3)
	Student	283 (52.2)
	Housewife	60 (11.1)
	Retired	24 (4.4)
	Other	3 (0.6)

* Lebanon, Egypt, Kuwait, Iraq, Qatar, Palestine, Kingdom of Saudi Arabia (KSA), Syria, and the United Arab Emirates (UAE).

### Shopping behavior during COVID-19 pandemic

As shown in Table 2, most study participants reported decreased shopping frequency (68.6%). On the other hand, the participants reported buying more amounts of food than usual (37.5%).

**Table 2:** Shopping behavior during COVID-19 pandemic among the study participants (n = 542).

Level	Response	n (percent)
**Shopping pattern (frequency of shopping)**	Shopping less than usual	372 (68.6)
	Shopping the same, I used to	120 (22.1)
	Shopping more than usual	50 (9.2)
**Amount of food purchased**	Buying less amount than usual	141 (26.0)
	Buying the same amount	198 (36.5)
	Buying more amount than usual	203 (37.5)
**Way of buying**	Ordering online	246 (45.4)
	Hypermarket	341 (62.9)
	Small markets or grocery store	355 (65.4)

### Food consumption behavior during COVID-19 pandemic

The majority of participants maintained their usual behavior concerning the consumption of food types, with a noticeable decrease in the consumption of fast food (47.6%) and eating in restaurants (69.2%), as shown in Table 3.

**Table 3:** Food consumption behaviors among the study participants (n = 542).

Behavior	Type	n (percent)		
		Less than usual	The same as usual	More than usual
Food Group	Fruits/vegetables	72 (13.3)	302 (55.7)	168 (31.0)
	Meat/ chicken	66 (12.2)	366 (67.5)	110 (20.3)
	Healthy food	100 (18.5)	299 (55.2)	143 (26.4)
	Fast food	258 (47.6)	158 (29.2)	126 (23.2)
	Canned food	143 (26.4)	294 (54.2)	105 (19.4)
	Sweets, cakes and pastries	113 (20.8)	235 (43.4)	194 (35.8)
	Frozen food	163 (30.1)	287 (53)	92 (17)
Eating between meals (Snacks)	Healthy snacks	106 (19.6)	338 (62.4)	98 (18.1)
	Unhealthy snacks	162 (29.9)	248 (45.8)	132 (24.4)
Eating pattern	Eating with family members	54 (10)	256 (47.2)	232 (42.8)
	Eating outside (restaurants)	375 (69.2)	130 (24)	37 (6.8)
Cooking pattern	Cook at home	42 (7.7)	253 (46.7)	247 (45.6)
	Ordering meals	205 (37.8)	168 (31)	169 (31.2)

Multiple linear regression of factors affecting restaurant dining revealed that participants’ gender was a significant variable (*p* <0.001). For females, 75.7% said that they eat less frequently in restaurants than usual, 18.4% said that their dining habits had not changed, and 5.8% reported an increase. In contrast, among males, 48.5% reported that they eat at restaurants less frequently than usual, 41.5% reported no change in dining, and 5.8% reported an increase.

### Food stored during the COVID-19 pandemic

Participants stored different food types, as shown in Table 4. Cereals, legumes, and their products were the most common food types held by participants (76.9%).

**Table 4:** Types of food stored by the participants during the COVID-19 pandemic.

Type of food	Yes	No
Cereals & legumes	417 (76.9)	125 (23.1)
Meat and chicken	371 (68.5)	171 (31.5)
Fish	153 (28.2)	389 (71.8)
Milk and dairy products	300 (55.4)	242 (44.6)
Canned food	357 (65.9)	185 (34.1)

Multiple logistic regression of factors affecting food storage during the home quarantine period during the COVID-19 pandemic among the study participants highlighted that only participants’ age significantly affected food storage, as shown in Table 5. Thus, being ≤ 25 years old (OR = 0.456, *p* value = 0.038) was significantly associated with storing more food than participants older than 25.

**Table 5:** Assessment of factors affecting food storage during the COVID-19 pandemic home quarantine period among the participants (n = 542).

Parameter	Food storage during COVID-19 pandemic home quarantine [0: No, 1: Yes]			
	OR	*p* value^#^	OR	*p* value^$^
**Country** • **Jordan** • **Other**	Reference 0.716	**0.194^**	0.734	0.236
**Gender** • **Female** • **Male**	Reference 0.964	0.873	----	----
**Age** • **≤ 25 years old** • **> 25 years old**	Reference 0.535	**0.002** ^**^**^	0.456	**0.038** *
**Educational level** • **< Bachelor degree** • **≥ Bachelor degree**	Reference 0.809	0.433	----	----
**Marital status** • **Married** • **Other (single, divorced, and widowed)**	Reference 1.761	**0.007^**	1.259	0.500
**Number of people living in the house** • **≤ six** • **> six**	Reference 0.776	0.262	----	----
**Profession** • **Still a student** • **Other (full-time, part-time, retired, etc.)**	Reference 0.747	**0.135^**	1.550	0.177

# Using simple logistic regression

$ Using multiple logistic regression

^ Eligible for entry in multiple logistic regression (significant at 0.25 significance level)

*Significant at 0.05 significance level.

### Feelings and emotions of participants during lockdown

The majority of participants suffered from one or more of the negative emotions and feelings. Feeling bored was reported the most (Table 6).

**Table 6:** Feelings and emotions that participants felt during the COVID-19 pandemic.

Emotion type	Yes	No
**Nervous**	333 (61.4)	204 (37.6)
**Worried**	401(74.0)	136 (25.1)
**Depressed**	358 (66.1)	182 (33.6)
**Sad**	343 (63.3)	193 (35.6)
**scared**	332 (61.3)	204 (37.6)
**Bored**	466 (86.0)	75 (13.8)

The negative feelings score had a mean of 3.22 (SD = 5.393), with a minimum score of -1 and a maximum score of 1. According to the multiple linear regression analysis of factors affecting the negative feelings score, as shown in Table 7, the country and gender were the significant factors. Therefore, participants in countries other than Jordan had a higher negative feeling score (Beta = 0.086, *p* value = 0.042). The negative feelings score for participants living in Jordan was 3.06 (SD = 5.375), while it was 4.10 (SD = 5.441) for other counties. Furthermore, females had a higher negative feeling score (Beta = -0.128, *p* value = 0.003) as the negative feelings score for females was 3.58 (SD = 5.443). On the other hand, it was 2.10 (SD = 5.091) for males.

**Table 7:** Assessment of factors affecting negative feelings score among the study participants (n = 542).

Parameter	Negative feeling score			
	Beta	*p* value^#^	Beta	*p* value^$^
Country • Jordan • Other	Reference 0.069	**0.107^**	0.086	**0.042** *
Gender • Female • Male	Reference -0.117	**0.006^**	-0.128	**0.003** *
Age • ≤ 25 years old • > 25 years old	Reference -0.122	**0.004^**	-0.088	0.242
Educational level • < Bachelor degree • ≥ Bachelor degree	Reference -0.017	0.691	----	----
Marital status • Married • Other (single, divorced, and widowed)	Reference 0.130	**0.002^**	0.109	0.110
Number of people living in the house • ≤ six • > six	Reference 0.013	0.764	----	----
Profession • Still a student • Other (full-time, part-time, retired, etc.)	Reference -0.070	**0.105^**	0.062	0.324

# Using simple linear regression

$ Using multiple linear regression

^ Eligible for entry in multiple linear regression (significant at 0.25 significance level)

*Significant at 0.05 significance level.

## Discussion

During the COVID-19 pandemic, Jordan and other countries have implemented numerous measures to restrain the invasion of SARS-CoV-2. One of these measures included lockdown for several weeks to months, alongside strict social distancing measures. These measures have impacted every sector in these countries, including education, the economy, and individuals’ mental and emotional health [9–11]. This online cross-sectional study affords a snapshot of the lifestyle habits among a sample of adults residing in Jordan and other Arab countries during the COVID-19 pandemic and the associated lockdown.

This study’s results showed that the participants’ dominant age group was 18–25 years. This aligns with the information provided in the Jordan statistical book (2016), which states that 62% of the Jordanian population is between 15 and 64 years old. Consequently, this particular age group is expected to be more accustomed to technology and online surveys than older individuals [12,13].

Shopping behavior was likely to change due to the rapid spread of COVID-19 and the associated lockdown that has imposed public fears and more stress on families. According to the study, approximately two-thirds of the participants reported decreased shopping frequency, buying more amounts, and storing certain types of food. Shopping behavior was likely to change due to the rapid spread of COVID-19 and the associated lockdown that has imposed public fears and more stress on families. It is thought that shopping can be one of the primary activities that increases the risk of contracting SARS-CoV-2 infection, consequently leading to decreased shopping frequency. This, in turn, increased the number of people shopping online [14]. Other reasons may be income limitations, reduced obtainability of goods, and partial access to food caused by restricted store opening hours [12,13]. Furthermore, participants noticed increased prices of certain foodstuffs such as meat, chicken, fruit, and vegetables. This could serve as another explanation for storing and buying more food.

The current study clearly reveals a decrease in ordering and buying food or eating at restaurants, demonstrating an increase in the rate of cooking and eating with family. Furthermore, it shows increased sugar consumption, fruits, vegetables, and unhealthy snacks.

Undoubtedly, the findings of the present study show the necessity for educational programs that encourage healthy eating and better buying habits. Because of their high amount of nutrients, it is well-known that eating a diet full of fruits and vegetables is essential.

These findings align with the results of Alhammouri and colleagues, who concluded that the intake of unhealthy food items such as fast food, uncontrolled eating, and snaking between meals increased significantly during COVID-19 [15]. Similarly, the Al-Hourani study, performed in Jordan among children and adolescents, and the Kolokotroni study, conducted in Cyprus, reported increased consumption of healthy and unhealthy food [7,9].

The results of the present study undoubtedly reveal the need for educational initiatives to improve shopping behavior and promote healthy eating choices. It is well-established that consuming a diet rich in vegetables and fruits is crucial due to their high content of phytonutrients, antioxidants, and anti-inflammatory compounds that might optimize the immunocompetence, which has been suggested to be beneficial in both the prevention and treatment of COVID-19 [16]. Although the COVID-19 crisis might have influenced shopping habits, food consumption habits, and mood, it is noteworthy that certain positive behaviors, such as consuming home-cooked meals and eating together with family members, have enlarged [15].

During the COVID-19 pandemic, people are exposed to negative feelings like anxiety, nervousness, worrying, depression, and fear. Different reasons can underline these negative feelings and moods, including the fear of contracting the disease, associated health consequences, the probability of isolation, financial demands, social media, and access to information and data [17,18].

It is necessary to learn from this experience to develop effective management strategies to overcome future risks of other pandemics. Among the suggested interventions is implementing online cognitive behavioral therapy (CBT) to provide mental health support. Additionally, involving mental health professionals and psychiatrists to guide the government on mental health policies is recommended to maintain a secure and trusted source of information [17,18]. According to the study’s findings, gender emerged as one of the significant factors affecting the negative feelings score, with females exhibiting higher scores than males. This aligns with another study conducted in 2020, which found that females had lower mental health scores [3]. This is also consistent with a survey conducted to assess gender differences in psychiatric symptomatology due to the COVID-19 pandemic and lockdown, which found that higher levels of anxiety and depression were found among females. These findings suggest that the variations in psychological responses between males and females may be attributed to the higher impact of the COVID-19 pandemic on women’s lives, as well as the differences in coping strategies and resilience based on gender [19].

### Limitations

The current study comes with limitations. First, research based on anonymous and online survey excludes the possibility of verifying the data on objective grounds. Hence, we encourage future research that can integrate additional empirical indicators to complement our findings. Furthermore, although our study aimed to capture a diverse range of perspectives, we recognize that the prevalence of students in our sample may limit the generalizability of our results to the broader adult population. Therefore, future research with a more balanced representation of various demographic groups, including a larger proportion of working adults, would provide a better understanding of puplic lifestyle habits during the pandemic. Finally, while the present study provides an overview of dietary habits and modifications during quarantine, its results cannot be interpreted in the context of long-term effects, as this was not an aim. All this suggests the need for cautious data interpretation and further studies in this field.

## Conclusions

The present study’s findings indicate that the global quarantine of the COVID-19 pandemic significantly impacted individuals’ shopping habits, dietary habits, and moods. It was observed that individuals tend to eat and snack more. Age, gender, and country of residency are among the factors that affect lifestyle habits. The COVID-19 pandemic has altered adults’ shopping, food consumption, and mood. Although positive behaviors have increased, such as shopping less frequently, eating home-cooked meals, and eating with family members, frequent snacking and storing food have been enlarged. Finally, public awareness of shopping and food consumption habits should be promoted.

## Supporting information

S1 Checklist (DOCX)

S1 File Supplementary file. (XLSX)
